# PACAP-38 in Acute ST-Segment Elevation Myocardial Infarction in Humans and Pigs: A Translational Study

**DOI:** 10.3390/ijms22062883

**Published:** 2021-03-12

**Authors:** Dora Szabo, Zsolt Sarszegi, Beata Polgar, Eva Saghy, Adam Nemeth, Dora Reglodi, Andras Makkos, Aniko Gorbe, Zsuzsanna Helyes, Peter Ferdinandy, Robert Herczeg, Attila Gyenesei, Attila Cziraki, Andrea Tamas

**Affiliations:** 1 Heart Institute, Medical School, University of Pecs, 7624 Pecs, Hungary; dora0szabo@gmail.com (D.S.); sarszegizsolt@gmail.com (Z.S.); nemeth.adam@pte.hu (A.N.); cziraki.attila@pte.hu (A.C.); 2 Department of Anatomy, MTA-PTE PACAP Research Team, Centre for Neuroscience, Medical School, University of Pecs, 7624 Pecs, Hungary; dora.reglodi@aok.pte.hu; 3 Department of Medical Microbiology and Immunology, Medical School, University of Pecs, 7624 Pecs, Hungary; polgar.beata@pte.hu; 4 MTA-SE System Pharmacology Research Group and Cardiovascular and Metabolic Research Group, Department of Pharmacology and Pharmacotherapy, Semmelweis University, 1089 Budapest, Hungary; saghy.eva@med.semmelweis-univ.hu (E.S.); makkos.andras@med.semmelweis-univ.hu (A.M.); gorbe.aniko@med.semmelweis-univ.hu (A.G.); peter.ferdinandy@pharmahungary.com (P.F.); 5 Pharmahungary Group, 6722 Szeged, Hungary; 6 Department of Pharmacology and Pharmacotherapy, Medical School, University of Pecs, 7624 Pecs, Hungary; zsuzsanna.helyes@aok.pte.hu; 7 Szentagothai Research Centre, University of Pecs, 7624 Pecs, Hungary; herczeg.robert@pte.hu (R.H.); gyenesei.attila@pte.hu (A.G.); 8 PharmInVivo Ltd., 7629 Pecs, Hungary

**Keywords:** pituitary adenylate cyclase activating polypeptide, acute myocardial infarction, ELISA, closed-chest myocardial infarction model, STEMI, cardioprotection, prognostic factor

## Abstract

Acute myocardial infarction (MI) is one of the most common causes of death worldwide. Pituitary adenylate cyclase activating polypeptide (PACAP) is a cardioprotective neuropeptide expressing its receptors in the cardiovascular system. The aim of our study was to examine tissue PACAP-38 in a translational porcine MI model and plasma PACAP-38 levels in patients with ST-segment elevation myocardial infarction (STEMI). Significantly lower PACAP-38 levels were detected in the non-ischemic region of the left ventricle (LV) in MI heart compared to the ischemic region of MI-LV and also to the Sham-operated LV in porcine MI model. In STEMI patients, plasma PACAP-38 level was significantly higher before percutaneous coronary intervention (PCI) compared to controls, and decreased after PCI. Significant negative correlation was found between plasma PACAP-38 and troponin levels. Furthermore, a significant effect was revealed between plasma PACAP-38, hypertension and HbA1c levels. This was the first study showing significant changes in cardiac tissue PACAP levels in a porcine MI model and plasma PACAP levels in STEMI patients. These results suggest that PACAP, due to its cardioprotective effects, may play a regulatory role in MI and could be a potential biomarker or drug target in MI.

## 1. Introduction

Despite modern interventional therapeutic options, acute myocardial infarction (MI) is still one of the most common causes of cardiovascular mortality and morbidity [1]. Cardiac biomarkers play an important role in the diagnosis of MI. Based on the latest recommendation of the European Society of Cardiology, the elevated high-sensitive cardiac troponin (hs-cTn) level prove the presence of myocardial injury, even though the elevated values do not represent the underlying cardiac or systemic pathologies [2]. The guideline highlights the complexity of the clinical circumstances making the differentiation difficult between ischemic and non-ischemic conditions associated with increased cTn levels [2]. Therefore, the latest studies have focused on the detection of potential new biomarkers having additional diagnostic or prognostic values to the routine parameters. Puelacher and co-workers tested the diagnostic accuracy of the combination of brain natriuretic peptide (BNP) and hs-cTn in patients with inducible myocardial ischemia and they did not certify any supplementary value of BNP in addition to hs-cTn [3]. In another study a biomarker risk score was defined with measurement of C-reactive protein, fibrin-degradation products and heat shock protein-70 in coronary artery disease patients to assess the risk of myocardial infarction or death [3,4].

To study the different underlying molecular processes of MI, several animal models are used for research. These preclinical studies—working with small or large animal MI models—have an important role in the development of potential new diagnostic and therapeutic approaches [5,6]. All these animal models have strengths and limitations. Large animal models have high translational value, as they show similarity in size, anatomy and physiology to human heart. In the last decade the catheter based closed-chest myocardial infarction large animal models have shown remarkable improvements in translational research, using clinically relevant interventional techniques and protocols [7]. In these models, reperfusion is performed after ischemia induction to examine the different biochemical changes after percutaneous coronary intervention, as MI treatment [8]. Moreover, different pre- and post- and remote conditioning manoeuvres were also studied to test their potential cardioprotective effects in pig [9,10] model.

Hypophysis adenylate cyclase activating polypeptide (PACAP), as a member of the vasoactive intestinal polypeptide (VIP)/secretin/glucagon family, is a ubiquitous, multifunctional neuropeptide [11]. PACAP acts on three different G protein-coupled receptors: the PAC1, VPAC1 and VPAC2 receptors, involving the cyclic adenosine monophosphate (cAMP)—and protein kinase A (PKA)-dependent signalling pathways [12]. PACAP exists in two biologically active forms, PACAP-38 and PACAP-27, containing 38 or 27 amino acids. PACAP-38 is the dominant form in mammals. In the last three decades numerous studies have examined the protective effect of PACAP in several ischemic diseases based on its antiapoptotic, anti-inflammatory and general cytoprotective effects [12,13,14], suggesting the applicability of PACAP as a potential cardiac biomarker, especially in pathologies with ischemic etiology [15,16,17,18]. Moreover, a general anti-ischemic effect of PACAP was also proved in the brain [19,20,21], in retinal diseases [22,23] and in different peripheral organs, like small intestine, kidney and liver [21,24,25,26]. Furthermore, PACAP type I receptor (PAC1 receptor) expression has already been confirmed in cultured cardiomyocytes, in mouse myocardium and also in human cardiac tissue [16,17,27,28,29]. Numerous in vitro studies have proven the cardioprotective effects of PACAP against ischemia and oxidative stress induced lesions [30,31]. Alston and co-workers examined sympathetic nerve fibers density and PACAP immunoreactivity in myocardial infarction mouse model. They found complete denervation in the infarcted region of the left ventricle. Moreover, they also detected significant elevated PACAP-38 immunoreactivity in the left ventricle free wall localized to the denervated infarct region. They found PACAP-38 immunoreactivity in extracellular matrix, myocytes and also in macrophages [32]. Furthermore, our research group established significant differences between the tissue or plasma PACAP levels of patients with different ischemic or non-ischemic cardiovascular diseases [29,33]. Based on all these results a general anti-ischemic protective role of this neuropeptide is presumable. Although the protective effect of PACAP against ischemic injuries is well know, there are no large animal models or human data available about plasma and tissue levels of PACAP in acute myocardial damages.

Therefore, our first goal was to examine tissue PACAP-38-like immunoreactivity (LI) in different myocardial (left ventricular, left and right atrial) tissue samples after three-hour or 72-h reperfusion in a clinically relevant, close-chest porcine model of reperfused MI. We compared the tissue PACAP levels of separate myocardial regions after MI and Sham operation and determined the effects of special pre- and postconditioning paradigms on tissue PACAP-38-LI alterations.

Our human study focused on determining plasma PACAP-38-LI in ST-segment elevation myocardial infarction patients before and after percutaneous coronary intervention (PCI) inducing revascularisation in comparison with controls. Correlation and multivariate analysis were performed with routine laboratory parameters, different drugs and echocardiographic dimensions to obtain more information about the potential cardioprotective effects of PACAP against ischemic injury.

## 2. Results

### 2.1. Changes of Tissue PACAP-38 Levels in Porcine Model of Acute Myocardial Infarction

**Figure 1:**
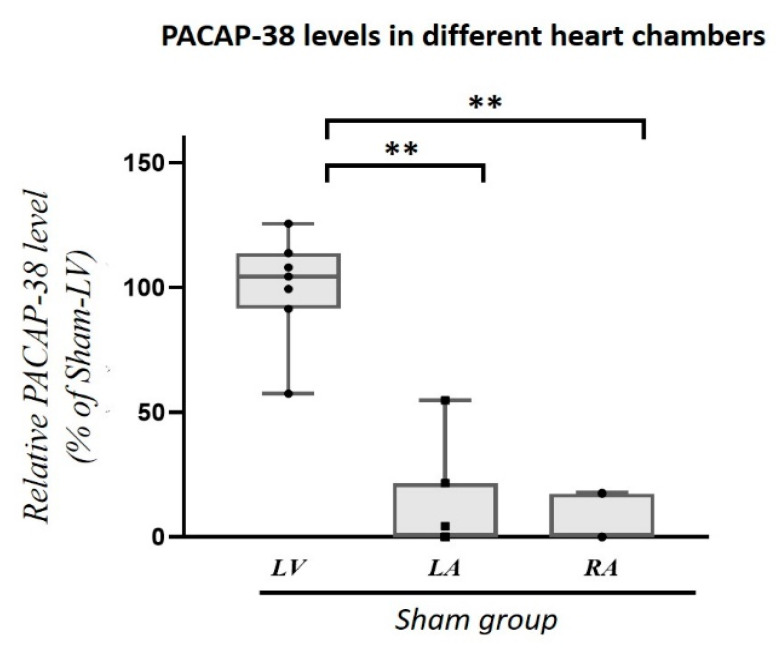
PACAP-38 levels of the different heart chambers (LV: left ventricle, LA: left atrium, RA: right atrium). In healthy, Sham-operated animals PACAP-38 level of the left ventricle (LV) was significantly higher than in left (LA) or in right atrium (RA). Data are expressed in ratio of Sham-LV PACAP-38 levels. The boxes show the interquartile ranges, and the whiskers indicate the most extreme observations. The middle line within the boxes represents the median value. Individual values are presented as black dots and squares. One-way ANOVA with Tukey post-hoc, ** *p* < 0.001 vs. Sham-LV group. *n* = 7–8.

To detect tissue level of PACAP-38, we examined the Sham-operated pig hearts without myocardial infarction (MI) in the left ventricle (LV) and left and right atrium (LA, RA). Significantly (*p* < 0.001) higher tissue PACAP-38 levels were detected in the left ventricle compared to both atria (Figure 1).

**Figure 2:**
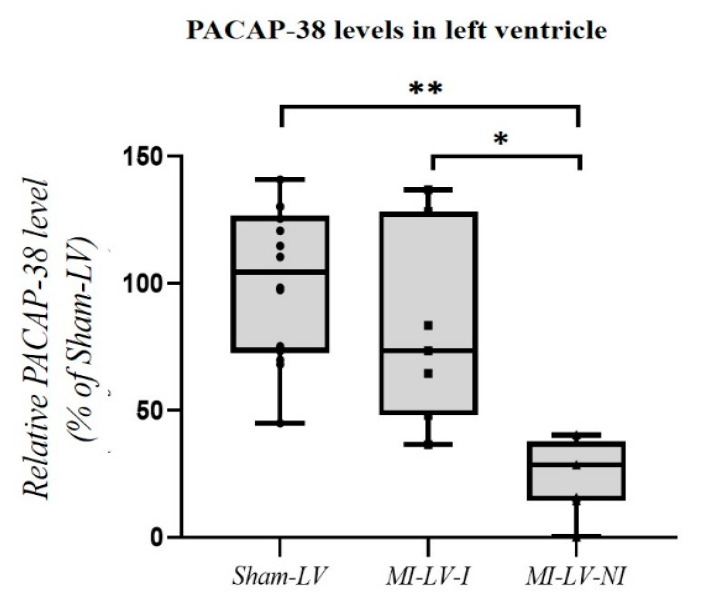
Differences between the tissue PACAP-38 levels in left ventricle in Sham-operated animals (Sham) and after myocardial infarction (MI) with time matched three-hour reperfusion. PACAP-38 level was significantly reduced in the non-ischemic left ventricular samples of MI hearts compared to the ischemic LV samples and also to the Sham hearts. Data are expressed in ratio of Sham-LV PACAP-38 level. The boxes show the interquartile ranges, and the whiskers indicate the most extreme observations. The middle line within the boxes represents the median values. Individual values are presented as black dots and squares. One-way ANOVA with Tukey’s post hoc test, * *p* < 0.05, ** *p* < 0.001 vs. Sham group. *n* = 7 (From each animal, two determinations were performed from 2 different region of the left ventricle (in case MI-LV: ischemic (MI-LV-I) and non-ischemic region (MI-LV-NI)/heart, in case Sham-LV: 2 regions equivalent to the MI-LV-I and MI-LV-NI regions/heart).

PACAP-38 level was measured in samples originated from different left ventricular (LV) regions in Sham and MI groups. Two samples from each animal were utilized, in case of MI group one of the samples was obtained from the ischemic region (MI-LV-I), while the other from the non-ischemic LV region (MI-LV-NI). In case of Sham group, we also collected two different samples from regions equivalent to the LV-I and LV-NI regions in MI hearts. We did not find significant differences between the two regions of the Sham-operated hearts (*p* = 0.902), thus, we did not separate them and we used one Sham group which contained all of the samples from the Sham heart. Although there was no significant difference between the relative PACAP levels in the ischemic LV samples (MI-LV-I) and the Sham hearts, the PACAP-38 level was significantly lower in the non-ischemic left ventricular samples of MI hearts (MI-LV-NI) compared to ischemic LV samples (*p* = 0.04) and also to the Sham hearts (*p* < 0.001) (Figure 2).

**Figure 3:**
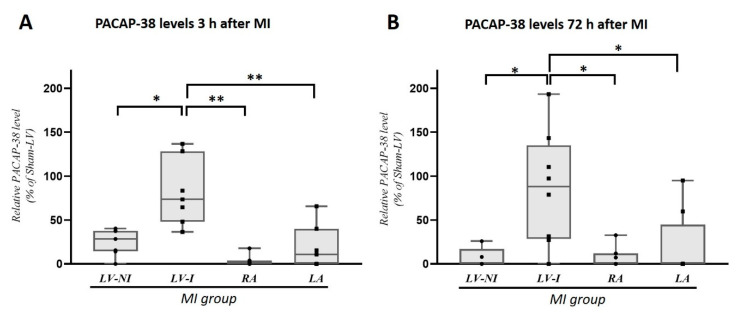
Comparison of the tissue PACAP-38 levels between different heart chambers and regions 3 h (**A**) or 72 h (**B**) after myocardial infarction (MI). PACAP-38 level was decreased in the non-ischemic region of the left ventricle (LV-NI) compared to the left ventricle ischemic region (LV-I) at 3 h after myocardial infarction (**A**). Significantly lower PACAP-38 levels were detected both in right atrium (RA) and left atrium (LA) compared to the ischemic LV region. Similar expression pattern was detected 72 h after myocardial infarction (**B**). The boxes show the interquartile ranges, and the whiskers indicate the most extreme observations. The middle line within the boxes represents the median values. Individual values are presented as black dots and squares. One-way ANOVA with Tukey post-hoc, * *p* < 0.05, ** *p* < 0.001 versus LV-I groups. *n* = 7–8.

In the MI porcine group PACAP-38 level was compared between different heart chambers and regions. Ischemic (LV-I) and non-ischemic (LV-NI) left ventricular samples and atria samples (RA, LA) were utilized after 3 h and after 72 h of reperfusion. Significantly lower tissue PACAP-38 levels were detected in the non-ischemic left ventricle (LV-NI) compared to the ischemic region (LV-I) after 3-h reperfusion (Figure 3A). PACAP-38 levels in both atria (RA, LA) were also significantly lower than in the ischemic left ventricular samples (LV-I). Similar expression pattern was found after 72-h reperfusion with lower values in LV-NI group and atrial tissues compared to LV-I samples (Figure 3B).

**Figure 4:**
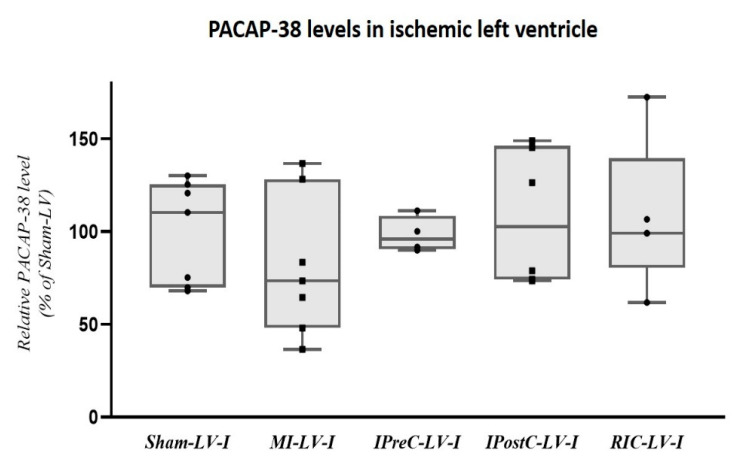
Differences between the tissue PACAP-38 levels of the Sham-operated (Sham), myocardial infarction (MI) alone and combined with ischemic conditioning methods: ischemic preconditioning (IPreC); ischemic postconditioning (IPostC); remote ischemic conditioning (RIC). There was no significant difference in PACAP-38 level in the ischemic zone of left ventricle between groups. The boxes show the interquartile ranges, and the whiskers indicate the most extreme observations. The middle line within the boxes represents the median values. Individual values are presented as black dots and squares. One-way ANOVA with Bonferroni’s post-hoc test. *n* = 4–8.

The impact of ischemic conditionings, which are cardioprotective manoeuvres, on the PACAP-38 level were also investigated (Figure 4). Left ventricular samples from the ischemic region were applied from MI and ischemic pre-, post- and remote conditioned animals. There were no significant differences between the 3 different conditioning methods (Figure 4).

### 2.2. Changes of Plasma PACAP-38 Levels in STEMI Patients

Sixteen STEMI patients (6 women, 10 men, mean age: 60.3 ± 2.96 years) and 12 controls (7 women, 5 men, mean age: 48.2 ± 5.34 years) were included in our human study. The main characteristics of the patients—risk factors, comorbidity, culprit coronary lesion, important echocardiographic parameters and the previous medication—are presented in Table 1.

In the control group we included patients with the symptoms of typical or atypical chest pain without any coronary lesion. The controls underwent elective coronarography examination showing healthy coronary without any significant stenosis or plaques. Seventy percent of these controls had musculoskeletal disorders and thirty percent of those had psychosomatic symptoms as underlying cause of the chest pain. The main characteristics of the patients, risk factors, comorbidity, and the previous medication are presented in Table 2.

**Figure 5:**
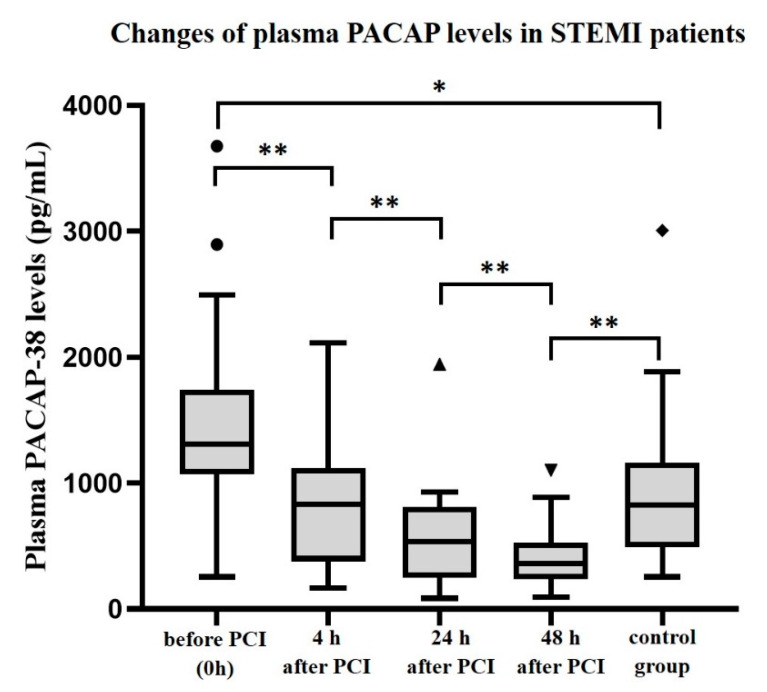
Changes of plasma PACAP levels in STEMI patient after PCI and in healthy control group. The solid bars represent medians of 16 patients and 12 controls. The boxes show the interquartile ranges, and the whiskers indicate the 10th and 90th percentile, outliers are also plotted with dots, rhombus and triangles. The middle line within the boxes represents the mean values. Wilcoxon Rank-Sum Test was used, statistically significant differences with *p*-values of ** < 0.001 and * < 0.05 are indicated.

Examining the plasma PACAP levels of the STEMI patients we found significantly higher levels before PCI, during the ischemic period, and a significant decrease of the plasma PACAP levels were detected right after PCI. Significantly (*p* < 0.001) lower PACAP levels were measured 4 h after PCI compared to the baseline values. The decline of PACAP levels was continuous during the examined period: We found significantly (*p* < 0.001) lower values 24 h after PCI compared to the 4 h samples. Moreover, there was a significant (*p* < 0.001) difference even between levels measured after 24 and 48 h (Figure 5). Differences between the plasma PACAP levels of the STEMI patients and the controls were also examined. We found significantly (*p* = 0.037) higher PACAP levels in 0 h samples of the STEMI patients compared to those of the controls (Figure 5). Furthermore, significantly (*p* = 0.009) higher plasma PACAP levels were detected in the control group compared to STEMI patients 48 h after PCI (Figure 5).

**Figure 6:**
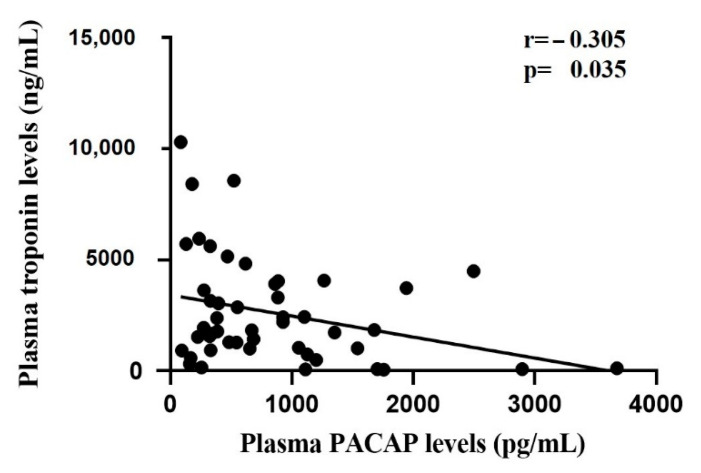
Correlation between PACAP and troponin levels in STEMI patients.

We found a significant (*p* = 0.035) weak negative correlation (r = −0.305) between all the time-matched plasma PACAP and troponin levels in the STEMI patient group (Figure 6). However, examining the time-matched PACAP and troponin levels separately (0, 4, 24, 48 h samples) there were no significant correlations (0 h: r = −0.191, *p* = 0.478; 4 h: r = −0.364, *p* = 0.301; 24 h: r = −0.185, *p* = 0.492, 48 h: r = −0.176, *p* = 0.513).

We also examined the different echocardiographic parameters related to the MI: ejection fraction, left ventricular end-diastolic diameter, the grade of mitral insufficiency and the number of the affected segments. There was no correlation between the echocardiographic parameters and the initial plasma PACAP levels, moreover, multivariate analysis did not detect any additive effects of the different parameters on the correlation with PACAP levels (Table 3).

Examining the connection between the main risk factors of STEMI (hypertension, diabetes mellitus, haemoglobin A1c (HbA1c) levels and smoking) and the initial PACAP levels, we found a significant (*p* = 0.034) positive correlation (r = 0.533) between hypertension and PACAP levels. However, there was no connection between the individual effect of the other risk factors and the examined polypeptide (Table 4).

**Figure 7:**
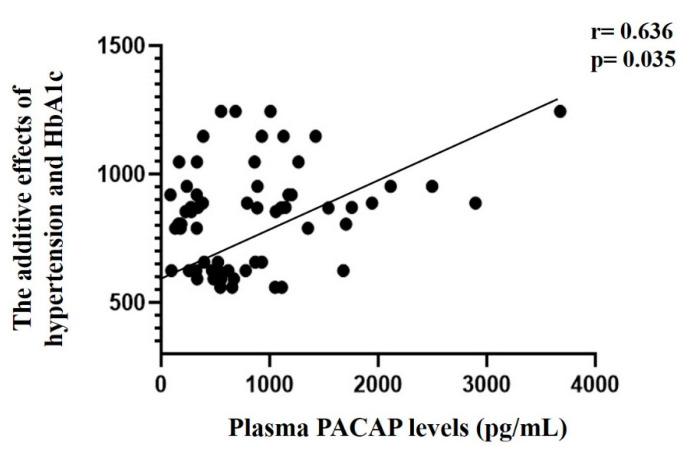
Connection between the additive impacting effect of hypertension and HbA1c and PACAP levels in MI patients.

Multivariate analysis showed a remarkable significant (*p* = 0.035) additive influencing effect (r = 0.636) of hypertension and HbA1c on the PACAP levels in MI patients (Figure 7). In contrast there were no individual or additive effects of hypertension and HbA1c on the correlation with plasma PACAP levels in the control group.

Examining the potential connection between the routine laboratory parameters (blood lipids: total cholesterol, low-density lipoprotein [LDL], high-density lipoprotein [HDL] and triglycerides [TG], C-reactive protein [CRP]) no significant correlation was found (Table 5) between the examined parameters and the initial plasma PACAP levels. Furthermore, multivariate analysis showed no additive effects of the laboratory parameters on the correlation with PACAP levels.

We also examined the previous anti-ischemic medication therapy of our patients. The correlation analysis detected no significant connection between these drugs and the initial PACAP levels (Table 6). Furthermore, multivariate analysis ruled out the potential additive effects of the different drugs on the PACAP levels.

## 3. Discussion

This study is the first translational demonstration of PACAP-38 level alterations after acute myocardial infarction and reperfusion in a porcine model and patients with STEMI.

Significantly, lower PACAP levels were detected in the non-ischemic region of the left ventricle in MI heart compared to the ischemic region and also to the Sham-operated left ventricle in porcine MI model. In STEMI patients, we found initially a significantly elevated PACAP-38 level in plasma samples before reperfusion. This significantly elevated PACAP level suggests a potential biomarker value of PACAP in case of acute ischemic myocardial lesions, similarly to other acute disorders or injuries, e.g., aneurysmal subarachnoid hemorrhage, spontaneous basal ganglionic hemorrhage and traumatic brain injury [13,34,35], where the authors also found significantly elevated PACAP levels after the lesions.

Hoover et al. detected the mRNA of PAC1 receptor in mouse heart, showing no significant regional differences between both atria and ventricles [27]. In contrast there are no experimental data about the potential differences of tissue PACAP-38 levels between the heart chambers. To answer this question, we examined first Sham-operated pig hearts without myocardial infarction. We detected significantly higher values in the left ventricle compared to the atrial tissues suggesting a LV-specific accumulation and/or local expression of PACAP-38. Based on these results, we focused on the left ventricle.

Examining the PACAP-38 level in the left ventricle of Sham and MI hearts significantly lower PACAP levels were detected in the non-ischemic regions of MI hearts, while the ischemic regions did not show significant differences from the Sham-LV samples. We assume that the two different MI-LV regions are not functionally independent, because of the extensive systemic effects of myocardial infarction (increased left ventricle filling pressure, sympathetic activation—increased catecholamine release, compensatory hyperkinesis of the non-ischemic regions, inflammatory and extracellular matrix processes) affecting the whole left ventricle [36,37,38]. Therefore, the changes in the different regions (LV-I and LV-NI) are strongly related to each other. One of the main sources of PACAP-38 in the tissue samples are the nerve fibers located between the myocytes [13]. Alston et al. showed that the sympathetic innervation density is decreased in the ischemic regions of MI-LV, compared to the peri-infarct region and Sham control hearts, on the other hand, they did not detect PACAP-38 immunopositive fibers in the peri-infarct region [32]. These data confirm our experiment, where we showed significantly decreased PACAP-38 level in the tissue samples of non-ischemic LV region. Although the nerve fibers are damaged in the ischemic region, we did not measure significantly lower level of PACAP-38 in the ischemic region of MI-LV compared to Sham samples. In contrast to the changes of nerve fibers, Alston et al. detected increased PACAP immunoreactivity in the extracellular matrix, myocytes and macrophages in the infarct region of mouse heart [32]. Based on these results we suppose that this increased PACAP immunoreactivity in the cells and the extracellular matrix could compensate the decreased PACAP level, caused by the nerve injury. In addition, PACAP is a well-established modulator of the immune system and depending on the immune status, disease and age, it may exert an anti-inflammatory role in different pathological conditions [39]. As acute myocardial ischemia induces an initial pro-inflammatory response that is followed by an anti-inflammatory phase to aid wound healing, scar formation and regeneration processes [40], we cannot exclude that ischemia/reperfusion induced injury might also trigger the local expression or deliberation/re-distribution of endogenous PACAP-38 and its receptors within the infarcted area. The transition between these two phases is orchestrated by finely regulated interactions between cardiomyocytes, endothelial cells, fibroblasts, and the interstitium as well as different players of both the innate and adaptive immune system. In our experiment, we examined homogenized heart tissue samples containing all the potential PACAP sources (macrophages, myocytes and nerve fibers), and because of the abovementioned compensatory mechanism, we did not find significant differences between the ischemic region of MI-LV compared to the Sham-LV heart samples.

In the last decades several preclinical studies have focused on different ischemic conditioning techniques with promising results for reducing myocardial ischemia or reperfusion injury after MI [41]. We measured tissue PACAP-38 levels of ischemic left ventricle after different ischemic conditioning approaches. There were no significant differences between the tested groups, which suggests that ischemic conditionings do not interfere with the PACAP-38 level in the infarcted myocardium in this porcine model. However, these results have a limited relevance due to the low case number in the different examined groups. Based on these animal experimental results we also performed a human study. There is only limited information about human PACAP levels in patients with heart diseases. In our earlier human study in patients with chronic heart diseases, significantly higher right auricle tissue PACAP-38 and PACAP-27 levels were detected compared to valvular diseases [29]. Moreover, earlier we found a significant negative correlation between plasma PACAP levels, and as a reliable prognostic marker of heart failure, an N-terminal pro B-type natriuretic peptide levels in chronic heart failure patients with dilated cardiomyopathy [33]. However, there were no data about the human plasma PACAP levels in MI.

In STEMI patients, plasma PACAP levels before PCI were remarkably higher compared to the healthy control group and significantly decreased after PCI. In contrast, the values 48 h after PCI were significantly lower than those of the controls. The significantly higher plasma PACAP levels in STEMI patients compared to the controls suggest that PACAP may have an important role in the pathomechanism of ischemic myocardial lesion and could be a potential biomarker in acute MI.

It is well-established that PACAP has a protective effect against oxidative stress-induced programmed cell death both in neuronal and non-neuronal cells. Earlier we examined the effects of PACAP treatment in neonatal rat cardiomyocyte cultures against ischemia/reperfusion(I/R)-induced apoptosis. PACAP treatment significantly increased cardiomyocyte viability and decreased apoptosis via the activation of PKA-Bad-14–3-3 and Akt-Bad-14-3-3 signalling pathways [30]. These results were also proved by Roth et al. who found that PACAP is protective against I/R injury involving inhibition of apoptosis signal-regulation, as well as caspase-3 activation and activation of phospho-Bad and Bcl-2 [31]. Based on the present clinical data we suppose that the main sources of PACAP are apparently the tissues known to contain the highest level of PACAP, such as brain and endocrine glands. In case of myocardial infarction, the increased PACAP level in the plasma and tissue samples additionally could originate from the nerve fibers, cardiomyocytes and macrophages [13,32]. It is also possible that the acute MI was preceded by several different ischemic attacks due to the atherosclerotic coronary arteries increasing the plasma PACAP level as the part of preconditioning. Furthermore, the remarkably decreased PACAP concentration after 48 h may represent the basal level of this neuropeptide suggesting that the lower basal PACAP level may be a predictive factor for acute myocardial infarction.

Examining the correlation between all the time-matched plasma PACAP and troponin levels revealed a significant weak negative correlation, although we did not find any significant connection between the time-related levels separately (0, 4, 24, 48 h). After PCI, a decrease of troponin levels is expected due to the revascularization. However, there is a high variability of serum troponin kinetics due to the different physiological (gender, age, race) and pathological (renal failure, pulmonary disorders, structural heart abnormalities) influencing factors, and the extent of the coronary occlusion (totally or intermittent occluded coronary artery—slow flow or no reflow phenomenon) [42,43,44]. Earlier studies proved that area under the curve (AUC) for elevated troponin is a useful method in the estimation of infarct size. It would require measurements of troponin level at six-to-eight different points in time at least. Since based on the latest therapeutic guidelines of MI, our patients were sent to an early active rehabilitation after 48 h of MI, the troponin measurement after 48 h was not feasible technically that resulted in the major limitation of our study. We believe that the high variability of serum troponin levels and the lack of the AUC calculation may explain the weak correlation between PACAP and troponin levels in our patients.

Examining the potential influencing effect of the different risk factors of MI on the plasma PACAP levels revealed no correlation between the presence of diabetes mellitus, smoking, elevated lipid levels and the examined polypeptide. However, a significant positive correlation was found between the initial PACAP levels and hypertension indicating that there may be an association between PACAP and one of the most important risk factors of STEMI. The results of the different in vivo animal experiments are conflicting about the connection between hypertension and PACAP. The presence of PACAP and PAC1 receptor in the cardiorespiratory sympathetic circuit suggests that this peptide may play an important role in blood pressure regulation [45,46]. Moreover, multivariate analysis showed a remarkably significant positive correlation between the additive effects of hypertension and HbA1c and the PACAP levels. This result suggests that hypertension and untreated diabetes mellitus together—representing a high risk for STEMI—are related to elevated PACAP levels. Diabetes mellitus is another main risk factor of MI, showing also interesting connection with PACAP. Several studies have explored the antidiabetic potential of PACAP due to the modulation of glucose-induced insulin secretion, the proliferation of islet cell mass, and increased glucagon response to insulin-induced hypoglycemia [47,48,49]. Moreover, several different in vivo animal experiments have already proved the important protective role of PACAP in diabetic nephropathy, retinopathy and vascular complication [50,51,52]. HbA1c is the most widely accepted marker for evaluating glycemic control thereby the therapeutic efficiency in diabetes mellitus. Despite of the positive additive effect of hypertension and HbA1c on the PACAP levels, we did not find any significant correlation between the individual effect of diabetes mellitus or HbA1c levels and the plasma PACAP levels in STEMI patients. Further human studies are needed to clear the exact influencing mechanisms of the different risk factors of MI on the plasma PACAP levels.

All of our STEMI patients had an echocardiographic examination on the second day according to the latest echocardiographic guidelines [53]. We did not find any significant correlation between the different echocardiographic parameters (LVEF, wall motion score index) and the initial PACAP levels. However, the early echocardiographic measurements can be misleading in reperfused patients due to the improvement of the left ventricular systolic function during the first 14 days [54,55]. Moreover, the recovery from stunned myocardium can be even more prolonged requiring four-to-six weeks [56].

We also examined the potential influencing effect of the different previous anti-ischemic medical therapies on the initial PACAP levels. More than 50% of our examined patients have already taken at least one anti-ischemic agent, though no significant correlation was detected suggesting that these drugs have no influencing effect on the plasma PACAP level. There are neither human nor experimental data about the different interaction between routinely used medical treatment and PACAP, further studies with large number of cases are needed to explore this question.

## 4. Materials and Methods

### 4.1. Animal Model

A closed-chest porcine model of reperfused acute myocardial infarction has been studied including 38 female pigs [9]. The following groups were applied:

- Sham-operated group (Sham): A balloon catheter was inserted in the left anterior descending (LAD) artery, but not inflated. Myocardial ischemia was not applied (*n* = 8).
- Myocardial infarction (MI) group: 90 min myocardial ischemia was induced with the occlusion of the LAD artery and followed by 3 h reperfusion (*n* = 7).
- Ischemic preconditioning group (IPreC): 3 × 5 min myocardial ischemia was applied before the 90 min LAD occlusion (*n* = 4).
- Ischemic postconditioning group (IPostC): 6 × 30 s myocardial ischemia was applied after the 90 min LAD occlusion, at the start of the reperfusion (*n* = 6).
- Remote ischemic conditioning group (RIC): 4 × 5 min hind limb ischemia was applied under the 90 min LAD occlusion; first cycle was applied 50 min after the beginning of the myocardial ischemia (*n* = 5).

**Figure 8:**
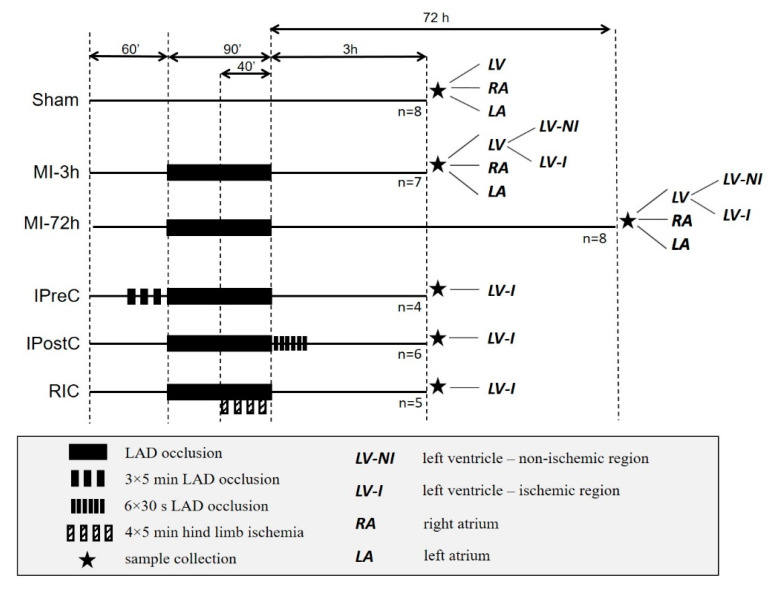
Groups of our clinically relevant, closed-chest porcine model of reperfused acute myocardial infarction. Sham: Sham-operated group; MI-3 h: myocardial infarction group with 3-h reperfusion; MI-72 h: myocardial infarction group with 72-h reperfusion; IPreC: ischemic preconditioning group; IPostC: ischemic postconditioning group; RIC: remote ischemic conditioning group.

Animals of each group were sacrificed after three hours of reperfusion and myocardial tissue samples were collected from ischemic and non-ischemic regions of the left ventricle (LV). The LV samples from the Sham hearts were collected from regions equivalent to the LV-NI and LV-I regions of MI hearts. The left and right atria (LA, RA) were also collected from Sham and MI group animals (Figure 8). In another series, MI group animals were sacrificed after 72 h (*n* = 8) and samples were collected from ischemic and non-ischemic region of the left ventricle, left and right atria (Figure 8).

The collected tissue samples were snap-frozen immediately after removal in liquid nitrogen, then ground and stored at −80 °C for cryopreservation until further use. Next, a total of 30 mg frozen cardiac samples were measured and sonicated on ice in 500 μL of ice-cold phosphate buffered saline (PBS) containing 14 μg of aprotinin as a protease inhibitor. A Hielscher UP 200 H/S homogenizer was used for sonication (Hielscher Ultrasonics GmbH, Teltow, Germany) with 3 × 30 s bursts and an amplitude of 30%. Then, the homogenates were centrifuged (10,000 rpm, 4 °C, 15 min) and the collected supernatants were further processed for PACAP-38 specific sandwich-type enzyme-linked immunosorbent assay (ELISA).

### 4.2. Human Study

Twenty patients with the diagnosis of ST-segment elevation myocardial infarction (STEMI) and 12 controls were enrolled into the present study. Patients were admitted to our clinic with the diagnosis of acute ST-elevation myocardial infarction on average 4–6 h after the beginning of the symptoms. At the hospital admission of the patients a 12-lead ECG and blood collection were executed and after routine physical examination coronarography was performed immediately. All patients had successful PCI with implantation of one or more drug eluting stents in one session. After the procedure, our patients were observed in the intensive care unit. According to our protocol every patient had blood collection four times during the examination period: first before the PCI (0 h), then 4, 24, and 48 h after PCI. During the first blood collection, three tubes [native, EDTA (ethylenediaminetetraacetic acid) and citrate tubes] were taken for hs-cTn and general laboratory testing. Inflammatory parameters (serum C-reactive protein level), kidney function parameters (serum creatinine and urea levels), complete blood count and lipid parameters (serum total cholesterol, LDL-cholesterol, HDL-cholesterol, and levels of triglycerides) were measured by the Department of Laboratory Medicine, University of Pecs.

**Figure 9:**
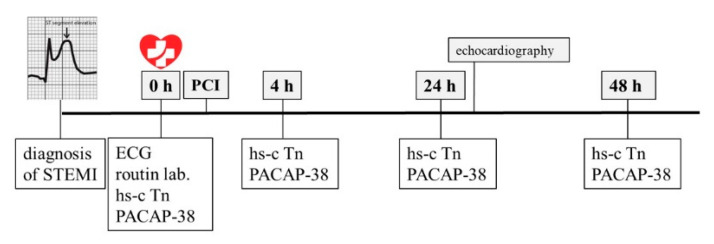
Human study protocol. STEMI: ST-elevation myocardial infarction, ECG: electrocardiography, lab.: laboratory examinations, hs-cTn: hypersensitive cardiac troponin.

For PACAP-38 determination, other 10 mL tubes of blood including EDTA were also taken. Because of the polypeptide nature of PACAP, a protease inhibitor (200 µL aprotinin (stock 1.4 mg/mL) into 10 mL blood) was added to the blood samples and an ice water bath was used for storing the tubes to avoid peptide degradation. The EDTA-tubes were centrifuged immediately after the collection (4000 rpm, 4 °C, 15 min), then the supernatant was collected and stored in polypropylene tubes (Sarstedt, Budapest, Hungary) at −80 °C until ELISA analysis. During the further three blood collections (4, 24, and 48 h after PCI) one native and one EDTA-tubes were taken for the determination of human hs-cTn and plasma PACAP levels. Echocardiographic examination was performed 24 h after the PCI to evaluate the left ventricular (LV) function, consequently the severity of the sustained MI and the additional MI complications using a high-quality portable echocardiograph (Philips Cx50—Philips, Amsterdam, The Netherlands). Routine two-dimensional (2D), M-mode, Doppler and Tissue Doppler measurements were performed. To define the LV ejection fraction (EF), the main characterized parameter of the LV systolic function the Simpson method was used (Figure 9).

Four patients were excluded from the study due to malignant ventricular arrhythmia followed by reanimation in two patients and pneumonia in further two patients. In the control group we included patients with the symptoms of chest pain without any coronary lesion. We collected blood samples from the control group similarly to the first blood test of the patient group: for routine laboratory examination and for PACAP detection. Without indication, we did not measure the high-sensitive troponin (hs-cTn) levels of our control group, presumably these values are lower than 14 ng/mL.

All human sample collections were carried out according to a protocol approved by the Institutional Ethic Committee (PTE KK 6383). In all cases, we obtained informed consent of the volunteers.

### 4.3. Measurement of PACAP-38-Like Immunoreactivity with ELISA Method

For determination of PACAP-38-like immunoreactivity (LI) in cardiac tissue homogenates and plasma samples sandwich-type enzyme-linked immunosorbent assay (human PACAP-38 ELISA kit, MyBiosource, Cat.No: MBS109020) was used according to the protocol provided by the manufacturer. PACAP-38-LI is referred to as PACAP-38 level in the manuscript.

Briefly, 50 μL of PACAP-38 standards, tissue homogenates and plasma of myocardial infarction patients and healthy controls were pipetted to the appropriate wells of the anti-PACAP-38 antibody-precoated microwells in duplicate. Then 100 μL of horseradish peroxidase (HRP)-conjugated reagent was added to each well, covered with closure plate, and incubated for 60 min at 37 °C. The plate was washed 4 times with 200 μL/well of 1× Wash buffer. Next 50 μL of Chromogen Solution A and 50 μL of Chromogen Solution B was added to each well and incubated for 15 min at 37 °C in dark. The developing colour reaction was stopped by adding 50 μL of Stop solution to every well. The SPECTROStar nano-spectrophotometer (BMG Labtech, Ortenberg, Germany) was used to measure the optical density (OD) of the test-wells at a wavelength of 450 nm. Since the obtained OD values were proportional to the level of PACAP-38 in the test samples, their concentrations were calculated by comparing the OD values of the sample wells to the ODs of the standard curve. All measured plasma PACAP-38 levels were demonstrated in pg/mL. Finally by the tissue samples the relative PACAP-38 levels were calculated by dividing the calculated PACAP-38 concentrations with the average PACAP level of the Sham-LV samples as control. The results were presented as % of control.

### 4.4. Statistical Analysis

For statistical analysis SPSS 21 (Statistical Package for the Social Sciences, Chicago, IL, USA) Program was used. In the first part of our study relative PACAP values (%) were used to eliminate the remarkable individual dispersion of porcine tissue PACAP levels. These values represented the percentage of the measured tissue PACAP-38 levels compared to the control sample (=the mean of the tissue PACAP-38 values in the Sham-operated LV samples). Kolmogorov-Smirnov and Shapiro-Wilk normality test were performed showing normally distributed data. To detect the potential differences between the examined groups One-way ANOVA with Tukey or Bonferroni’s post hoc tests were used. In our human study after Kolmogorov-Smirnov normality test Friedman and post-hoc test were performed to detect the changes of plasma PACAP levels after PCI in STEMI patients. To examine the differences between plasma PACAP levels of STEMI patients and controls Wilcoxon Rank-Sum Test was used. The interaction between PACAP and hs-cTn, EF and other potential impacting factors were tested with Spearman’s correlation. Based on the correlation coefficient (the r value) we could define positive (r = 0–1) and negative (r = −1–0) correlation including subgroups with different strength. Multivariate regression analysis was performed to examine the additive effects of the main influencing factors. In all cases *p* < 0.05 was considered statistically significant.

## 5. Conclusions

This study first reports about the tissue PACAP-38 levels in a relevant, large animal MI model. Its significantly lower levels in the non-ischemic region of the left ventricle in MI animals can be explained by the lack of PACAP-38 immunopositive nerve fibres. Although the nerve fibers are damaged in the ischemic region, we detected similar PACAP-38 levels in the ischemic region of MI-LV compared to the Sham-LV heart samples, because the increased PACAP immunoreactivity in the myocytes, macrophages and the extracellular matrix could compensate the decreased PACAP level caused by the nerve injury. The first human study showed—in agreement with earlier results—elevated initial plasma PACAP levels in STEMI patients compared to the healthy controls assuming an acute protective role of the peptide against ischemia. Furthermore, the significant decrease in plasma PACAP levels after successful revascularisation also confirms this theory, since after the termination of acute ischemia, there is no more need for acute cardioprotective factors. Summarizing the significant changes in plasma PACAP levels we suggest that this peptide, due to its anti-ischemic and cardioprotective effects, plays an important protective role in acute myocardial infarction. Further examinations are necessary to ascertain whether PACAP might be a potential prognostic biomarker of MI in the future.

## Figures and Tables

**Table 1:** Main demographic and clinical characteristics of the patients.

Number of patients	16
Smoking	50% smoker, 12.5% former smoker
Hypertension	56.25%
Diabetes mellitus	25%
Hyperlipidemia	31.25%
Obesity	43.75%
Known ischemic heart disease	12.5%
Culprit coronary lesion	left anterior descending artery (LAD)—31.25%
	circumflex coronary (Cx)—12.5%
	right coronary artery (RCA)—56.25%
Previous medication therapy	ACE inhibitor/ARB—62.5%
	ß receptor blockers—43.75%
	platelet aggregation inhibitors—43.75%
	statin/fibrate therapy—43.75%
	antianginal medications—18.75%
Cardiac function (echocardiographic parameters)	left ventricular ejection fraction (LVEF): 46.19% (±10.6)
	left ventricular end-diastolic volume (LVEDV): 135.12 mL (±33.8)
	left ventricular end-systolic volume (LVESV): 71.81 mL (±25.8)

**Table 2:** Main demographic and clinical characteristics of the controls.

Number of controls	12
Smoking	8.3% smoker, 0% former smoker
Hypertension	33.3%
Diabetes mellitus	16.7%
Hyperlipidemia	33.3%
Obesity	25.0%
Previous medication therapy	ACE inhibitor/ARB—25.0%
	ß receptor blockers—16.7%
	platelet aggregation inhibitors—16.7%
	statin/fibrate therapy—0%
	antianginal medications—0%

**Table 3:** Correlation between the different echocardiographic parameters and the plasma PACAP levels.

Echocardiographic Parameters	Correlation Coefficient (r Value)	Significance (*p* Value)
ejection fraction (EF—%)	r =− 0.059	*p* = 0.829
LV-EDD (mm)	r = 0.308	*p* = 0.246
mitral insufficiency (I-IV grade)	r = −0.095	*p* = 0.726
wall motion score index (WMSI)	r = 0.250	*p* = 0.926

**Table 4:** Correlation between the main risk factors of STEMI and the plasma PACAP levels.

The Main Risk Factors of STEMI	Correlation Coefficient (r Value)	Significance (*p* Value)
hypertension	r = 0.533 *	*p* = 0.034
diabetes mellitus	r = −0.157	*p* = 0.563
haemoglobin A1c (HbA1c)	r = 0.201	*p* = 0.456
smoking	r = −0.143	*p* = 0.598

* statistically significant, *p* < 0.05.

**Table 5:** Correlation between the routine laboratory parameters and the PACAP levels.

The Routine Laboratory Parameters	Correlation Coefficient (r Value)	Significance (*p* Value)
Total cholesterol	r = −0.127	*p* = 0.640
LDL	r = −0.015	*p* = 0.957
HDL	r = 0.100	*p* = 0.712
TG	r = −0.027	*p* = 0.922
CRP	r = 0.163	*p* = 0.546

**Table 6:** Correlation between the previous medication therapy and the PACAP levels.

Previous Medication Therapy	Correlation Coefficient (r Value)	Significance (*p* Value)
ACE inhibitor/ARB	r = 0.252	*p* = 0.346
ß-blockers	r = −0.254	*p* = 0.342
Platelets aggregation inhibitor	r = −0.039	*p* = 0.130
Statin/fibrate therapy	r = −0.308	*p* = 0.246
Antianginal medications	r = 0.041	*p* = 0.880

## Data Availability

The data presented in this study are available in the article, there is no supplementary data.
